# Long Non-Coding RNAs in Infection Biology

**DOI:** 10.3389/fgene.2012.00308

**Published:** 2013-01-09

**Authors:** Vinod Scaria, Ayesha Pasha

**Affiliations:** ^1^GN Ramachandran Knowledge Center for Genome Informatics, Institute of Genomics and Integrative Biology, Council of Scientific and Industrial ResearchDelhi, India; ^2^Open Source Drug Discovery Unit, Council of Scientific and Industrial ResearchNew Delhi, India

**Keywords:** long non-coding RNA, infection, pathogen, immune, host-pathogen interactions

## Abstract

Long non-coding RNA have emerged as an increasingly well studied subset of non-coding RNAs (ncRNAs) following their recent discovery in a number of organisms including humans and characterization of their functional and regulatory roles in variety of distinct cellular mechanisms. The recent annotations of long ncRNAs in humans peg their numbers as similar to protein-coding genes. However, despite the rapid advancements in the field the functional characterization and biological roles of most of the long ncRNAs still remain unidentified, although some candidate long ncRNAs have been extensively studied for their roles in cancers and biological phenomena such as X-inactivation and epigenetic regulation of genes. A number of recent reports suggest an exciting possibility of long ncRNAs mediating host response and immune function, suggesting an elaborate network of regulatory interactions mediated through ncRNAs in infection. The present role of long ncRNAs in host-pathogen cross talk is limited to a handful of mechanistically distinct examples. The current commentary chronicles the findings of these reports on the role of long ncRNAs in infection biology and further highlights the bottlenecks and future directions toward understanding the biological significance of the role of long ncRNAs in infection biology.

## Background

Recent large-scale, high-resolution transcriptome analyses of several vertebrate genomes, including that of humans, has confirmed the transcriptional potential of large tracts of the vertebrate genome and further opened up new vistas in the study of transcriptomics, RNAs, and their subclasses (Lee and Schatz, 2012). One recent aspect of transcriptomics which has notched up immense interest amongst researchers is the class of RNA’s which do not have the potential to code for proteins. This class of RNA’s have been dubbed as the non-coding RNAs (ncRNAs) and a subset of this class are the long ncRNAs (long ncRNAs). This recently characterized class (Hung and Chang, 2010) is composed of longer (>200 nt) transcripts and encompasses a number of previously annotated classes of non-coding transcripts such as antisense, lincRNA, processed pseudogenes, and others (Kapranov and St Laurent, 2012). The general features which have been established for long ncRNAs includes their being encoded by RNA Polymerase II and that are capped, polyadenylated like protein-coding transcripts. However, recent reports, including one from our group, suggests a distinct epigenetic regulation of long ncRNAs in comparison to protein-coding genes (Sati et al., 2012). The present estimates for the number of long ncRNAs has crossed 18,000 loci, and extends to cover a number of functionally and mechanistically distinct sub-types.

Despite the scientifically proven functional relevance of long ncRNAs, only a few of them have been well characterized and studied for their roles in the regulation of gene expression. Further, only a handful of articles published to date study the mechanism through which these ncRNAs exert their critical role in several biological scenarios such as genomic imprinting, epigenetic modification, and post-transcriptional regulation of genes. However, given their relevance within the biological machinery as well as hard to ignore significance numerous conceptual mechanistic approaches have been presented to explain their functionality. These range from their possible interaction with other biomolecules within cells to processing into smaller regulatory RNAs (Jalali et al., 2012) and several others (Wang and Chang, 2011).

In comparison to long ncRNAs, most of the RNA subclasses within the ncRNA family have been extensively studied with respect to their roles in viral pathogenesis, antiviral functions, host-pathogen cross talk, immuno-regulatory functions, etc. (Scaria et al., 2006; Rederstorff and Hüttenhofer, 2010). Only a few reports cover the role of long ncRNAs in infection biology inspite of their implicated role in several disease processes such as cancer, psoriasis, Crohn’s disease, and many others. Given the significant role of these RNAs in biomolecular regulatory interactions within cells and their prospects as drugs targets in numerous diseased conditions, it becomes highly pertinent and imperative to perform an exhaustive evaluation of their functionalities so as to provide a clear picture of their role in the cellular regulatory machinery and disease processes (Yan and Wang, 2012).

Although the study of the role of long ncRNAs in infections has come a long way from the preliminary studies on viral transcript interactions with cellular components (Keene, 1985), the growth rate in the understanding of long non-coding RNA functionality in infections has not been proportionate to that for other RNAs. The present commentary chronicles the role of long ncRNAs in infection biology, with special focus on integrating evidence of their functional role within the same at multiple levels as well as suggesting the potential way forward in examining and unraveling the distinct role of long ncRNAs in several aspects of infection, immunity and host-pathogen cross talk.

## Long Non-Coding RNA Characterization, Biogenesis, Processing, and Function

The initial set of long ncRNAs were identified and characterized from the EST libraries present for humans and other model organisms such as Mouse, owing to the persistent and large-scale initiatives such as the H-Invitational Database (Yamasaki et al., 2010) and the FANTOM consortium (Ravasi et al., 2010). Following this was the annotation of the transcriptional potential of the genome through tiling microarrays (Kapranov et al., 2003), that provided deep insights into the hidden transcriptional potential of the genome. Furthermore, the recent availability of high-throughput sequencing techniques and transcriptome analysis upto single nucleotide resolution, have added to the number of annotated non-coding RNA loci. The present Gencode annotation includes over 18,000 long ncRNAs. It is generally perceived that most of the long ncRNAs are transcribed by RNA Pol II, with few exceptions. While lncRNAs are known to be essential components of epigenetic gene expression modulation, there exists a limited amount of data on the epigenetic regulation of lncRNA. However, a recent study has pointed toward the similarity of epigenetic regulation of lncRNAs to that of mRNA except for its dissimilarity with regard to DNA methylation (Sati et al., 2012). Further evidence from our group highlights the origin of several novel and previously functionally characterized small RNA from lncRNAs and in most cases from the ends of lncRNAs (Jalali et al., 2012). These results point out to the antecedent of functional small RNA clusters from lncRNA and hence, the potential regulatory role of long non-coding RNA in biological realms. The functions of long ncRNAs are distinct and modulated through biomolecular interactions with other cellular moieties that have been reviewed in detail by Lipovich et al. (2010) and participate in a wide spectrum of regulatory interactions ranging from antisense regulation to epigenetic modification.

## Long Non-Coding RNA Expression in Response to Infection

Recent reports have increasingly suggested the potential functional consequences of long ncRNAs in infection biology owing to the dysregulation of these ncRNAs during infection processes mostly in response to viral pathogens. One study by Saha et al. (2006) highlighted the role of NEAT1, previously annotated as the Virus Inducible non-coding RNA (VINC1), in the mouse brain following infection with the Japanese Encephalitis virus. Followup studies on the same long non-coding RNA have emphasized the role of NEAT1 in paraspeckle formation and has been further speculated to be a key component in the host responses to viral infections such as the Japanese Encephalitis. Similarly another long non-coding RNA, namely, Psoriasis susceptibility-related RNA Gene Induced by Stress (PRINS) has been shown to be upregulated following infection with the Herpes Simplex Virus (Sonkoly et al., 2005) and treatment with bacterial cell wall extracts (Bari et al., 2011) apart from other stress factors such as ultraviolet radiation, etc. Unbiased genome-wide associations have also implicated PRINS as a conspicuous factor in psoriasis (Sonkoly et al., 2005). Further, a recent report by Peng et al. (2010) has indicated distinct signatures of long non-coding RNA expression in a SARS infection model and significant dysregulation in response to infection. Similar responses were also obtained post-treatment with interferons, indicating the possibility of a common pathway for infection response and long non-coding RNA regulation mediated through the interferon gamma involved immunological pathways. The role of long non-coding RNA in infections has not been restricted solely to animals but also extended to those in plants and model systems like Dictyostelium. In the model organism *Dictyostelium discoideum*, a long non-coding RNA DutA (Development-specific but UnTranslatable RNA A) has been determined to be dysregulated post-infection (Farbrother et al., 2006), while in plant systems, the PINCI1 family has been identified to be upregulated following *P. infestans* infection in potato (Avrova et al., 2007).

## Long Non-Coding RNAs and Immune Cells

One of the major determinants of infection biology in higher organisms is an intact immune system, the absence of which marks an immunocompromised condition. Several studies have been conducted to date with the aim of unraveling the role of non-coding RNA regulatory networks in the overall infection process, particularly those which are related to microRNAs. Recent reports have seen widespread attention on long non-coding RNA based regulation in immune cells and immunological responses. Pang et al. (2009) utilized sequencing based transcriptome analysis to characterize the transcriptome and determine the number of long ncRNAs that are expressed in CD8+ T-cells. Additional in-depth analyses also suggested that many of these long non-coding RNA loci share their genomic loci with protein-coding genes, with some of these overlapping small RNA sites, thereby indicating the possible mechanism through which the processing of a subset of long ncRNAs takes place. An example of the role of long non-coding RNA in immunological function is that of TMEVPG1 (Theiler’s murine encephalomyelitis virus persistence candidate gene 1) which has been shown to be expressed post-infection in the CD4 and CD8+ T-cells and downregulated following immune cell activation (Vigneau et al., 2003) possibly through the regulation of IFN gamma.

## Evidence from Genome-Wide Associations

The development of unbiased genome-wide assays for genetic markers associated with several traits has contributed immensely to the understanding of the mechanizations underlying the role of long ncRNAs in disease processes. Several studies have utilized genome-wide associations to enunciate the relationship of long non-coding RNA loci with progression to Hepatocellular Carcinoma following infection with Hepatitis. One of these studies has identified the genomic SNP rs7763881, mapping to HULC loci, as causative of conferring decreased susceptibility to Hepatocellular carcinomas in HBV (Liu et al., 2012) while another has indicated four SNPS in 8p12, which map to a long non-coding RNA loci, as conferring increased susceptibility to infection in certain patients (Chan et al., 2011). Two similar studies for HIV have indicated an endogenous retroviral locus that codes for a long non-coding RNA as a genetic loci for susceptibility to infection with HIV (Dalmasso et al., 2008; Limou et al., 2009).

## Pathogen Encoded Long Non-Coding RNAs

Pathogen genomes have also been confirmed to encode for long ncRNAs with several viruses encoding for long ncRNAs. This includes the EBER1 and EBER2 RNAs encoded by the Epstein–Barr Virus that have been determined to be associated with the particular viruses latency and malignancy (Nanbo et al., 2005; Iwakiri et al., 2009; Iwakiri and Takada, 2010); beta 2.7 RNA encoded by the Cytomegalovirus, which prevents the host cell from undergoing apoptosis following infection and ensuring cell survivability conducive to the virus (Reeves et al., 2007; Zhao et al., 2010); HSUR1 and HSUR2 encoded by the Herpesvirus genome, that mimics the target for the microRNA mir-27 and hence, modulates the T-cell gene expression (Cook et al., 2005; Buck et al., 2010; Cazalla et al., 2010) and lastly, VAI and VAII coded for by the human adenovirus and implicated as key determinants in the suppression of host cell-derived RNAi response following viral replication (Andersson et al., 2005; McKenna et al., 2006; Sano et al., 2006; Xu et al., 2007). In a peculiar case, as exemplified in the case of HBZ encoded by HTLV-1, increasing evidence suggests apart from coding for a protein, the mRNA could have non-coding functions (Chaudhary and Ratner, 2011; Nakano and Watanabe, 2012) with distinct nuclear retention and possible effect in persistence and immune evasion (Rende et al., 2011). Apart from viruses, long ncRNAs have been also identified in the pathogen, *Plasmodium falciparum* wherein a genome-wide transcriptome annotation by Broadbent et al. (2011) showed the presence of 22 Telomere-associated ncRNAs. These lncRNAs were found to be localized to the perinuclear compartment of the pathogen (Sierra-Miranda et al., 2012) and contributing to its biology by possible interaction with nuclear proteins.

## Long Non-Coding RNAs in Host-Pathogen Interaction

**Figure 1:**
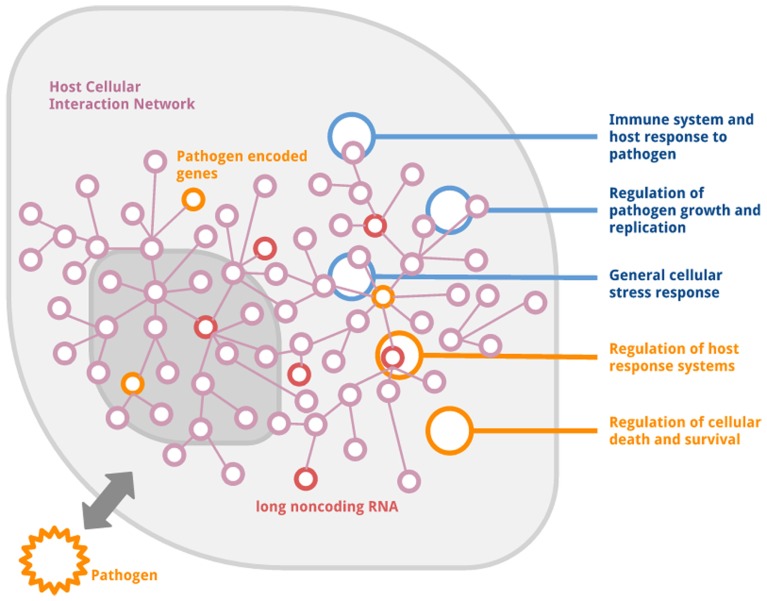
**Schematic of the role of long non-coding RNAs in host-pathogen interaction networks**.

At present the mechanistic understanding of the role of lncRNAs in or following infection and host responses to infection may at best be described as being sketchy and limited to a few studies and even more so, primarily to four models only. Each of these reports presents an interesting new opportunity to understand and evaluate the function and interaction of long non-coding RNA following infection. Mechanistically, dysregulation of long ncRNAs could modulate downstream regulation of genes at several functional levels ranging from epigenetic changes influencing chromatin organization to post-transcriptional regulation at transcript levels as well as through direct interaction with other biomolecules such as proteins and RNAs (Wang and Chang, 2011; Moran et al., 2012). These interactions could modulate (a) host responses to an invading pathogen not excluding immunological mechanisms (b) regulation of pathogen growth and replication (c) regulation of cellular death/apoptosis or survival (d) general stress responses. While considerable contention exists over the exact mechanism through which viral lncRNAs act, it has been suggested that the viral long ncRNAs potentially exploit the interaction networks within hosts, thereby affecting their response to infections in an attempt to evade the immunological onslaught. They do this through a variety of mechanisms, including the inhibition of RNAi response or target mimicry (Franko-Zorilla et al., 2007). The schematic overview of the reported mechanisms through which long ncRNAs mediate in host-pathogen interactions has been summarized in Figure 1.

## Genome-Scale Biology and Opportunities in Understanding Role of Long Non-Coding RNAs in Infection and Host Response

Recent technological advancements in the analytical techniques for transcriptomic evaluation has opened up new vistas in the possibilities through which the function, interaction, and regulatory mechanisms of lncRNAs may be understood at the genomic level. Further developments in computational methodologies have enabled researchers to functionally characterize lncRNA through the interpretation and analysis of genome-scale transcriptome correlations. These improvements have been aided in part from the generation and availability of large central repositories containing data pertinent to large-scale experiments which may be utilized to integrate datasets with the aim of understanding long non-coding RNA biology. Recent reports on the integration of datasets on disparate transcriptomes have provided an immense impetus to the understanding of functional consequence of long ncRNAs in cancers (Brunner et al., 2012). These interpretations could be further extrapolated to evaluate specific signatures and hence identify the long ncRNAs that are involved in infections and host immune-responses consequent to infection. Finally, the availability of similar technologies to understand protein-RNA interactions (Yeo et al., 2009; Hafner et al., 2010; Wen et al., 2011; Ascano et al., 2012; Scheibe et al., 2012) and epigenetic modifications during infection processes also opens up vast new opportunities in the understanding of the biology and regulation of long ncRNAs during infection.

The conclusions derived from the studies conducted to date as well as further evaluations would provide novel approaches for implementation in drug development and discovery through the design of targeted strategies that would specifically aim biomolecular interactions mediated by long ncRNAs.

However, the specific targeting of RNAs using small molecules would necessitate an accurate estimation of RNA structure that would in part be aided by recent techniques such as SHAPE (McGinnis et al., 2012) and PARE (Yu et al., 2007) that offer attractive possibilities in the understanding of RNA structure for larger lengths of transcripts. Recent studies have highlighted the distribution of specific regulatory motifs like G quadruplexes in RNA, which may serve as specific targets for RNAs (Biffi et al., 2012; Weng et al., 2012).

## Future Perspective

Based on the literary evidence to date, it is apparent that lncRNAs play a significant role in the overall infection and immunological process. Several viral lncRNAs such as EBER1, EBER2, VAI, VAII, etc., have been positively ascertained to play a significant role in increasing the pathogen survivability within the host. While on the other hand, numerous RNAs present in humans have also been identified to play a critical role in host immunological responses (such as the TMEVPG1, HUCL, etc.) influencing the overall outcome of infections depending upon their ability to either increase or decrease patient susceptibility to infections (Vigneau et al., 2003; Dalmasso et al., 2008; Limou et al., 2009; Chan et al., 2011; Liu et al., 2012). In view of the consequential role of lncRNA in infection biology, it is imperative to completely elucidate their complete function and regulatory role post-infections and analyze the bottlenecks within. This has in part been aided by the recent leaps in transcriptome technology and data generation, however, there is still an immense scope within the field for the same. Recent studies by Amit et al. (2009) and Shapira et al. (2009) provide interesting insights into the role of RNA machinery and factors involved in host-pathogen interaction following infection. While the former puts forward an unbiased approach for elucidating the mechanisms through which regulatory networks control transcriptional response post-infections, the latter explores the host-pathogen factors and pathways involved through which the H1N1 virus exerts its effect on host systems subsequent to infection. Once understood, these lncRNAs may serve as drug targets that may be utilized for further drug development as well as to study the host-pathogen interplay following infection, thereby increasing the possibility of improving patient prognosis post-infection.

## Conflict of Interest Statement

The authors declare that the research was conducted in the absence of any commercial or financial relationships that could be construed as a potential conflict of interest.
